# Assessment of Factors Affecting the Implementation of Integrated Management of Neonatal and Childhood Illness for Treatment of under Five Children by Health Professional in Health Care Facilities in Yifat Cluster in North Shewa Zone, Amhara Region, Ethiopia

**DOI:** 10.1155/2019/9474612

**Published:** 2019-12-14

**Authors:** Ayele Mamo Abebe, Mesfin Wudu Kassaw, Fikir Alebachew Mengistu

**Affiliations:** ^1^Department of Nursing, Debre Birehan Health Sciences College, Debre Birhan P. O. Box 37, Amhara Region, Ethiopia; ^2^Department of Nursing, Woldia University, Woldia Town, Amhara Region, Ethiopia; ^3^Department of Nursing, Dessie Health Sciences College, Dessie, Ethiopia

## Abstract

**Background:**

Every year some 12 million children in developing countries die before they reach their fifth birthday. Seven in ten of these deaths are due to acute respiratory infections (mostly pneumonia), diarrhea, measles, malaria or malnutrition. The WHO Department of Child and Adolescent Health and Development (CAH), in collaboration with eleven other WHO programmes and UNICEF, has responded to this challenge by developing the Integrated Management of Childhood Illness (IMCI) strategy. Research that examines assessment of factors influencing the implementing the integrated management of neonatal and childhood illnesses (IMCI) strategy in Ethiopia is limited.

**Objective:**

To assess factors influencing the implementation of the IMNCI strategy by health professionals in public health institutions of Yifat cluster in North Shewa zone, Ethiopia, 2018.

**Method:**

An institutional based cross-sectional study will be conducted from March to May. A total of 201 health professionals will be selected using proportionally allocated to population size and interviewed using structured and pretested questionnaires. Data will be coded, entered and cleaned using SPSS version 20 for analysis. Univariate (frequency), Bivariate, Multiple logistic regression analysis will be employed. *P*-value and 95% confidence interval (CI) for OR will be used in judging the significance of the associations. *P*-value less than 0.05 will be taken as significant association.

**Results:**

Data were obtained from 201 health care professionals, yielding a response rate of 100%. The overall IMNCI implementation was 58% as high level implementation and 42% as low level implementation. In multivariate analysis the implementation of IMNCI was higher among IMNCI trained health care professionals ([*AOR* = 2.7, 95% CI: (1.1.278, 4.562)]) and among those whose always referring chart booklet [*AOR* = 2.76, 95% CI: (1.753, 5.975)].

**Conclusion:**

IMNCI strategy can be better implemented through provision of training for the health workers. However, a variety of factor found to be a barrier to IMNCI implementation in a consistent way. Recommendations have been made related to provision of the training to the nurses and Health Care system strengthening among others.

## 1. Introduction

### 1.1. Background

The Integrated Management of Neonatal and Childhood Illness (IMNCI) strategy is an integrated approach to child health that holistically focuses on the well-being of the whole child. The IMCI strategy aims to reduce illness, disability, and death, and to promote improved growth and development among children under 5 years of age. The strategy includes both preventive and curative elements that are implemented by families, communities, and health care facilities [1–3]. Implementation of integrated management of childhood illness (IMCI) in comprehensive and holistic approach that forms bench mark for basic child health in promoting celebration of fifth birth day for children below five years of age, free from Malaria, Pneumonia, Diarrhea, Measles and Malnutrition [2–4]. The Integrated Management of Childhood Illness (IMCI) remains the cornerstone for child survival strategies and for improving the quality of care provided to sick children in health facilities in over 100 countries [4]. Every year some 12 million children in developing countries die before they reach their fifth birthday. Seven in ten of these deaths are due to acute respiratory infections (mostly pneumonia), diarrhea, measles, malaria or malnutrition. The WHO Department of Child and Adolescent Health and Development (CAH), in collaboration with eleven other WHO programmes and UNICEF, has responded to this challenge by developing the Integrated Management of Childhood Illness (IMCI) strategy [2, 5]. The main objectives of the strategy are to reduce death and the frequency and severity of illness and disability, and to contribute to improved growth and development [5].

The Integrated Management of Childhood Illness (IMCI) remains key strategy to reducing child mortality. The strategy includes improving case management skills of sick children at first level health facilities, strengthening health system and improving family and community to promote child health. In Africa region, 22 countries are now implementing IMNCI in over 75% of districts, compared with only 10 countries in 2007 [6]. Ethiopia has one of the highest under-five mortality rates with more than 321,000 children under the age of five dying every year. As children usually present with more than one of the preventable disease conditions, it was recognized that there was need for an integrated approach in order to manage the child in a holistic manner. This led to the development of IMNCI strategy which integrates all available measures for health promotion, prevention and integrated management of childhood diseases through their early detection and effective treatment, and promotion of healthy habits within the family and community [7].

The importance of having an Integrated Management of Newborn and Childhood Illness strategy is that it enables a consistent and standardized approach that addresses the major causes of under-five morbidity and mortality which are responsible for more than 90% of the mortality in this age group in Ethiopia [2, 7]. The strategy includes three main components: Improvements in the case-management skills of health staff through the provision of locally adapted guidelines on IMCI and through activities to promote their use. Improvements in the health system required for effective management of childhood illness. Improvements in family and community practices [8]. The first and second international consultant training on IMNCI implementation were conducted in Addis Ababa in November 1995 and May 1996 respectively, using semi-adapted IMNCI training materials for Ethiopia. The Federal Ministry of Health (FMOH) selected three regions for initial implementation: Tigray, Southern Nation, Nationalities and Peoples Region (SNNPR) and Addis Ababa. The first and second national IMNCI review and re-planning workshops were conducted in March 2001 and 2004 respectively. All regions have included IMCI in their annual health plan of action and are actively implementing IMNCI [9].

### 1.2. Statement of the Problem

Several studies conducted in different countries indicated that the implementation of the IMNCI strategy is still inadequate. These studies also mentioned a variety of factors that influences the implementation of the strategy by health workers. The most common identified problems are lack of training, poor supervision, lack of IMNCI essential drugs and on jobs aid, health workers perception, shortage of the staffs, nature of the strategy and lack of support from the government and stake holders [10–21]. Every day about 16,000 under five children continue to die in 2015, most of them will perish from preventable causes, such as pneumonia, diarrhea and malaria [22]. Annually 1.12 million neonatal deaths occur in the African region which accounts quarter of all under five deaths. Half of these deaths occur in just five countries-Ethiopia, Nigeria, Democratic Republic of Congo, United Republic of Tanzania and Uganda. Saving these lives would take and estimated extra US $1.39 per capita per year. It is also estimated that every minute eight under five children die in sub-Saharan Africa. Two third of this deaths occurs due preventable cause of deaths such as pneumonia 21%, malaria 18%, diarrheal diseases 16%, measles 5% and HIV/AIDS 6% most of which complicated by malnutrition that accounts one third of all deaths in children under five years [23]. Recent reports indicate that neonatal mortality, infant mortality and under-5 mortality stand at 37, 59 and 67, respectively per 1000 live births [24, 25]. Although Ethiopia has achieved Millennium Developmental Goal MDG 4 targets of reducing child mortality and witnessed a steady reduction in child mortality (more than 40%) across the country, over 300,000 children under the age of five still die each year from preventable or treatable conditions such as diarrhea, ARI primarily pneumonia, and malaria, neonatal problem in combination with malnutrition [25, 26]. Health facility surveys carried out in three regional states of Ethiopia: Amhara, Oromia and Southern Nations and Nationalities Peoples' (SNNP) indicated low coverage of IMCI in the regions. IMCI coverage was 20% for Amhara, 4% for Oromia, and 25% for SNNP Region. The percentage of trained targeted health workers in the three regions was even lower than the IMCI coverage. The proportion of under-five cases assessed by IMCI trained health workers ranged from 0% in Oromia, to 16% in Amhara and 32% in SNNP region [27]. Regarding the factors that influences IMNCI implementation this survey also revealed that Consultation time was lower than recommended by IMCI guidelines (15–20 minutes) although it was higher for IMCI trained health workers (13 minutes in average). Supplies were available for most health facilities but there was inadequate supervision. The integrated index of assessment was on average 0.42 of the recommended 10 variables. Most children were not checked for general danger signs, nutritional status or vaccination. There was over classification of pneumonia and under classification of malaria in all three regions and the percentage of children treated correctly was low [27]. A variety of efforts has been made by the government of Ethiopia In attempt to overcome this problem and further reduce child mortality rate. For example trying to increase the cumulative number of Health centers (HC) providing Integrated management of neonatal and childhood illness and training Health Extension Workers (HEWs) on the issues to manage childhood illness at community level, however the implementation of the IMNCI strategy may get hampered by many challenges [26, 28, 29]. Multi-country survey on global challenges with scale up of the IMNCI revealed that the perceived challenges in implementing the IMNCI strategy in Ethiopia are Long duration of training, Prohibitive financial regulations, Inadequate Funds for supervision, training, printing, refreshments, videos, and also Lack of material, transport, clinical instructors [30]. To best of the researcher knowledge, there are no sufficient research reports on factors influencing implementation of the Integrated Management of Neonatal and Childhood Illnesses (IMNCI) strategy in Ethiopia. The researcher believes that the scarcity of available data on this topic in the study area has limited the development of intervention strategies in line with SDG. Hence, this study is aimed at assessing factors influencing IMNCI implementing by health professionals in Yifat cluster, North Shewa, Amhara region, Ethiopia.

### 1.3. Significant of the Study

During this time, the IMNCI strategy has an important role in reduction of child mortality and morbidity. In Ethiopia the study conducted on the factors influencing the implementation of the IMNCI strategy is not yet documented. Therefore this study seeks to assess the factors influencing the implementation of IMNCI strategy by the nurses in in Yifat cluster.

Therefore, firstly the data from this study will be useful to health planners such as those responsible bodies working on integrated management of neonatal and child hood illness and will also enable such bodies to design better programs to address the identified problems. Secondly the study findings will be useful for the community in reduction of under-five mortality and morbidity. Lastly, this paper will be used as important literature for future researchers who want to undertake similar study.

## 2. Literature Review

### 2.1. Introduction

This section presents the result and the idea taken from different studies that was reviewed from different literature including journals, articles and other literature related to this topic that deals with factor influencing implementation of Integrated management of neonatal and childhood illness by health workers in health institutions.

### 2.2. IMNCI, Neonatal and Child Health

Annual number of deaths among children less than 5 years of age has decreased by almost a third over the last three decades though the reduction is not similarly distributed around the world. In developing countries over 10 million children die every year before they reach their fifth birth day. Seven in 10 of these deaths are due to acute respiratory infections (mostly pneumonia), diarrhea, measles, malaria, or malnutrition — and often to a combination of these illnesses [31]. As indicated in 1996 analysis unless greater efforts are made to control childhood illnesses they will continue to be major contributors to child deaths through the year 2020. Due to this assumption that the new strategies to significantly reduce child mortality and improve child health was introduced [2, 32]. During the mid-1990s, the World Health Organization (WHO), in collaboration with UNICEF and many other agencies, institutions and individuals, responded to this challenge by developing a strategy known as the Integrated Management of Childhood Illness (IMCI) which is composed of preventive and curative interventions that aims to improve practices and the quality of management of childhood illness linking the programs, such as immunization, nutrition, control of malaria and other infectious disease in health facilities, the health system and at home to be implemented in an integrated manner [8, 33]. IMNCI adopts an algorithmic approach that encourages health providers to address a sick child in a systematic manner to address several medical conditions, that often coexist, rather than the presenting symptom only which often is the case when child health programs are implemented in a vertical fashion. Apart from treating medical condition, the strategy insists that each contact with the child can be utilized for preventive and promotive health interventions [34]. Ethiopia adopted the Integrated Management of Neonatal and Child hood illness strategy in 1996 with the aim of reducing the unacceptably high child hood mortality and morbidity and to promote child health and development [9, 35].

### 2.3. Global Burden of Neonatal and Child Deaths

Child mortality is a core indicator for child health and well-being including health and nutrition status. It is also a key indicator of the coverage of child survival interventions and, more broadly, of social and economic development [36]. World leaders agreed on the millennium developmental goals (MDGs) in 2000 and called for reducing the under-five mortality rate by two thirds between 1990 and 2015 known as the MDG 4 target [37]. In June 2012, world leaders renewed their commitment during the global launch of Committing to Child Survival. A Promise Renewed, aiming for a continued post-2015 focus to end preventable child deaths. With the end of the MDG era, the international community is in the process of agreeing on a new framework — the Sustainable Development Goals (SDGs). The proposed SDG target for child mortality represents a renewed commitment to the world's children: By 2030, end preventable deaths of newborns and children under five years of age, with all countries aiming to reduce neonatal mortality to at least as low as 12 deaths per 1,000 live births and under-five mortality to at least as low as 25 deaths per 1,000 live births [37, 38]. The world has made substantial progress in improving child survival in the past 25 years. The global under-five mortality rate dropped 53 (50, 55) percent, from 91 (89, 92) deaths per 1,000 live births in 1990 to 43 (41, 46) in 2015). Globally, the annual number of under-five deaths dropped from 12.7 million to 6 million in 2015. Despite substantial gains, progress is insufficient to achieve the MDG 4 target yet and the progress has not been enough, and the target risks being missed at global level [36, 37, 39].The global under-five mortality rate needs to be reduced to 29 deaths per 1,000 live births which implies an annual rate of reduction of 14.2 percent for 2011–2015, much higher than the 2.5 percent achieved over 1990–2011 [40]. At the regional level, all MDG regions except Oceania have more than halved the under-five mortality rate. Eastern Asia, Latin America and the Caribbean, and Northern Africa have reduced the under-five mortality rate by two thirds or more since 1990. At the country level, about a third of countries (62) have reduced their under-five mortality by two thirds or more and achieved the MDG 4 target set in 2000. Among them are 12 low-income countries including Ethiopia and another dozen middle income countries and an additional 74 countries reduced their under-five mortality rates by at least half and another 41 countries by at least 30 percent [37]. According to UNICEF (2015) reports that from all the under five deaths, almost 45 percent occur in the neonatal period due to many complications. Globally, infectious diseases, prematurity and complications during labour and delivery are the main causes of death for children under age 5. Infectious diseases account for about half of global under-five deaths. Of the 5.9 million underfive deaths in 2015, almost half were caused by leading infectious diseases and conditions such as pneumonia, diarrhea, malaria, meningitis, tetanus, measles, sepsis and AIDS.7 globally, the main killers of children under age 5 in 2015 were preterm birth complications (18 percent), pneumonia (16 percent), intrapartum-related complications (12 percent), diarrhea (9 percent) and sepsis/meningitis (9 percent) [41].

### 2.4. Regional Child Mortality

Accelerating progress in child survival urgently requires greater attention to ending preventable child deaths in Sub-Saharan Africa and Southern Asia. Under-five deaths are increasingly concentrated in Sub-Saharan Africa and Southern Asia, while the share in the rest of the world dropped from 32 percent in 1990 to 18 percent in 2013.Though Sub-Saharan Africa has seen the decline in the under-five mortality rate accelerate, with the average annual rate of reduction increasing from 0.8 percent in 1990–1995 to 4.2 percent in 2005–2013, the region still has the highest child mortality rate 92 deaths per 1,000 live births, more than 15 times the average for developed regions. By 2050 close to 40 percent of all births will take place in Sub-Saharan Africa, and 37 percent of children under age five will live there, so the number of under-five deaths could stagnate or even increase without more progress in the region [36]. Sub-Saharan Africa has the highest risk of death in the first month of life and is among the regions showing the least progress. About half of under-five deaths occur in only five countries: India, Nigeria, Democratic Republic of the Congo, Pakistan and China. [40, 42]. Among the ten MDG regions, sub-Saharan Africa contributed roughly half (49.6%, 3.113 million) of under-5 deaths worldwide in 2013, and southern Asia almost a third (32.1%, 2.015 million [43]. The leading causes of death among children under age five are pneumonia (18% of all under-five deaths); preterm birth complications (14%); diarrhea (11%); intrapartum related complications (complications during birth; 9%); and malaria (7%). Globally, more than a third of under-five deaths are attributable to under nutrition [36, 38].

### 2.5. Child Mortality in Ethiopia

In 1990, the under-five mortality rate for Ethiopia was one of the highest in the world at 204 per 1,000 live births. Nevertheless, by 2011 and 2013, according to 2011 EDHS findings and the 2013 United Nations Inter Group for Mortality Estimation (UN IGME), with an average annual rate of decline of 5.0%; this rate was reduced to 88 and 64 per 1,000 live births, respectively. With an under-five mortality rate of 64, Ethiopia has attained MDG4. Despite this great achievement, the trend in neonatal mortality remained stagnant and contributed a large proportion (nearly half) of the under-five mortality rate [44, 45]. According to the recent World Health Statistics Report published in 2014, Ethiopia has achieved MDG 4 three years earlier by reducing under-five mortality from 1990 estimate. The Proportion of children who were dying before their fifth birthday has declined to 64 in 2013 from 204 in 1990 among 1000 live births [22]. The annual number of under-5 deaths in Ethiopia has been reduced by more than half, from an estimated 412,000 deaths in 2000 to 196,000 in 2013. In the period from 2000 through 2011, an estimated 469,000 child deaths were prevented through high-impact child health interventions, nearly ¾ of them due to interventions that were scaled up after 2005 Major disparities in mortality rates and coverage show that many of Ethiopia‘s poorest children, and those living in rural areas and remote regions, are still excluded from essential care, and the equity gaps are getting worse. While mortality rates have improved for children in the wealthiest 80% of households, children in the poorest 20% are being left behind [44]. Several factors across multiple sectors have played a key role in driving the progress in child health. Reductions in child mortality are associated both with improved coverage of effective interventions to prevent or treat the most important causes of child mortality in particular essential immunizations, malaria prevention and treatment, vitamin A supplementation, birth spacing, early and exclusive breast feeding — and with improvements in socioeconomic conditions [45].

### 2.6. IMNCI Implementation and Influencing Factors

The study conducted in India revealed that poor supervision and monitoring, in adequate availability of essential drug while another study conducted in Kenya found that economic, social, cultural, type of facility and in adequate stakeholders support are the major factors that affect the implementation of IMCI strategy [12, 13]. The facility survey conducted in China showed that few sick children were fully assessed, and only 43.8% were correctly classified by health workers and the use of antibiotics for sick children was high and not according to guidelines [15]. According to the study conducted in Benin 63.6% children were treated in accordance with IMNCI guidelines and Performance of individual health workers varied greatly, from 15–88% of patients treated correctly, on average and also important performance gaps found immediately after IMCI training persisted due to inadequate supervision and monitoring [10, 21]. Similar study conducted in Kenya found that utilization and implementation of IMCI concepts in the region is approximately 14% which is below standard level established (above 68%), by WHO and UNICEF, due to recurrent staff deficit and also limited resource at disposal from the region [13]. Results from multi country evaluation indicated that IMCI case management training is an effective intervention to improve the rational use of antimicrobial drugs for sick children visiting first-level health facilities in low-income and middle-income countries. In another way IMCI trained health workers are significantly more likely prescribing correct treatment than no IMCI trained workers [14]. Similar another study done in Benin also revealed that health workers with training plus study supports performed better than those with training plus usual supports (20.4 and 19.2 percentage point improvements for recommended treatment [*p* = 0.08] and —recommended or adequate” treatment [*p* = 0.01], respectively. Increased supervision frequency was associated with better care (odds ratio for recommended treatment = 2.1 [95% confidence interval: 1.13.9] per additional supervisory visit [18]. In Ghana the IMNCI implementation remained poor despite many efforts made by the stakeholders to improve the quality the utilization. The main reasons for poor implementation of the IMNCI strategy are the frontline health workers perception toward the strategy and work over load [17]. Another study conducted in Tanzania found the factors that affect health workers Adherence to IMCI protocols are poor supervision practice, shortage of facilitator and funds, reluctance to refer facility layout frequent rotation of staff within facilities and high attrition rates, feature of the strategy, lack of IMCI drugs and job aids [19]. Similar study conducted in lower resource setting country indicated that IMCI-trained workers were more likely to correctly classify illnesses (*RR* = 1.93, 95% CI: 1.66–2.24). Workers with lower baseline performance showed greater improvements in prescribing medications (*RR* = 3.08, 95% CI: 2.04–4.66), vaccinating children (*RR* = 3.45, 95% CI: 1.49–8.01), and counseling families on adequate nutrition (*RR* = 10.12, 95% CI: 6.03–16.99) and administering oral therapies (*RR* = 3.76, 95% CI: 2.30–6.13) after training and supervision [20]. The study conducted at community health center of Indonesia found several factors that influences IMNCI implementations including shortage of health workers trained in IMCI which were only 43% of health center had all health workers in the child care unit trained in IMCI and 40% of health center conducted on the job training. Regarding health care system supplies, only 19% of Health center (HC) had all the essential drugs and equipment for IMCI. In another way lack of perceptions of IMCI benefits, Lack of supervision from district health office staff and low community awareness regarding the importance of IMCI were also reported [12, 46, 47]. The survey conducted in Afghan and south Africa identified that short duration of training, lack of ongoing follow up and clinical supervision, high cost of training, lack of political support; lack of human and material resources and time for IMCI implementation, poor reading ability of health workers, and mismatch between training needs and resources available, frequent health worker turnover and poor quality of IMCI implementation by those trained specifically in the use of job aids and protocols for assessment, classification, treatment and counseling are some challenges to implement IMNCI strategy [48, 49]. Multi country survey on global challenges with scale up of IMCI strategy mentioned political, resource or cost related and strategic related and other challenges with their sub contents as follows, lack of buy in from regional stakeholders 45.2% and competing priorities 22.6% are political challenges, while cost and human resource related factors are expensive 74.2%, inadequate fund for printing modules 38.7%, inadequate fund 38.7%, the other challenges , long duration of course 51.6%, lack of clinical materials16.1% and lack of transport 19.4% [30]. Another study conducted in Zambia and Rwanda showed that Staff turn-over, stock-outs, lack of time due to inadequate staffing, lack of equipment, a work overload and unequal distribution of professional nurses on duty per shift, poor knowledge, lack of privacy in the facility, Human Immune Deficiency Virus HIV related stigma from both health workers and care givers and other facility/systems gaps were identified as barriers to IMCI implementation [50–52]. According to the study done in Republic of Tanzania there are three reasons contributing to health workers' non-adherence to IMCI guidelines; these are the use of single, narrow diagnoses rather than IMCI classifications; the belief that chloramphenicol is unacceptably toxic, and the belief that referring severely ill children is often unnecessary [53]. Review of systematic challenges to the scale-up of integrated community case management in six African countries including Ethiopia, Ghana, Malawi, Mali, Mozambique, and Niger reported that the common challenges to scale-up of intervention packages for child survival in particular the delivery approach of integrated community case management (ICCM) are; the deployment, supervision, motivation and retention of community health workers as the backbone of ICCM, maintaining reliable supply chains, demand side barriers to utilization, weak monitoring and evaluation systems, and the need for supportive government policies and engagement to achieve sustainable progress [54]. In several countries health worker often mentioned the increased time required for a consultation and the difficulty of following the IMCI guidelines when there is a high patient load. Additionally Staff turnover, low staff motivation due to low salary (Uganda), and Poor supervision, lack of funds for travel, hospital related expenses are also mentioned. Another challenges identified in Bangladesh, Cambodia, Niger, Cambodia, and Zambia and Uganda are low use of public sector health care for a variety of reasons (accessibility, official or under-thetable user fees, perceived poor quality, lack of drugs, and so on), lack of separate budget for strategy, Conflict between IMCI guidelines and existing policies and regulations(Soviet republics of Kazakhstan and the Kyrgyz Republic, Morocco [55, 56]. A primary Health Care Nurses (PHCNs) of selected clinic of Limpopo Province in Rwanda categorizes the difficulties of rendering IMNCI into lack of resources and poor working conditions. lack of human resources, lack of material resources, shortage of medication, Absence of computer at clinics and lack of physical resources are subcategorized under lack of resources while the lack of support by the supervisors, burn out related to lack of support by stake holders, lack of cooperation from community members, lack of support from media, lack of political support and sign and symptoms related to work overload are subcategorized as poor working conditions which have negative impact on the nurses [57]. In another way the problems identified in pre service IMNCI training are lack of political commitment, lack of supervision, availing training materials, shortage of tutors, problems in curricula integration and shortage of IMNCI teaching and learning materials, shortage of trained teachers and shortage of IMNCI learning materials were reported [19, 58, 59].

**Figure 1:**
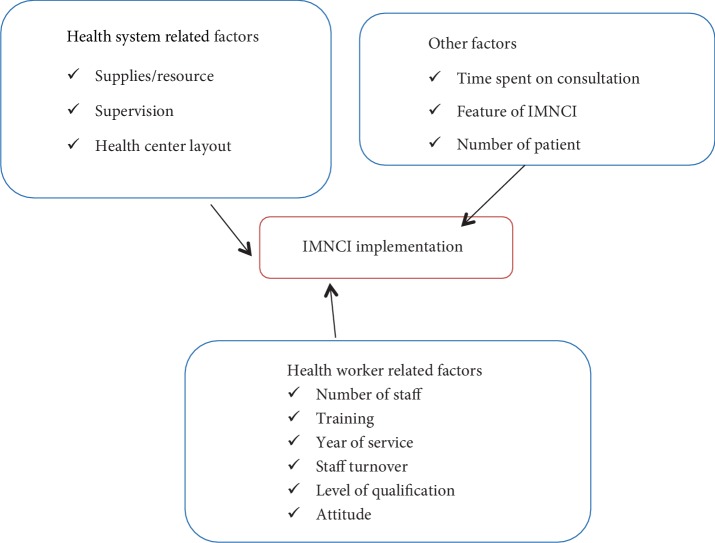
Conceptual framework on factors that influencing IMNCI implementation, adopted by the researchers from different literatures.

**Figure 2:**
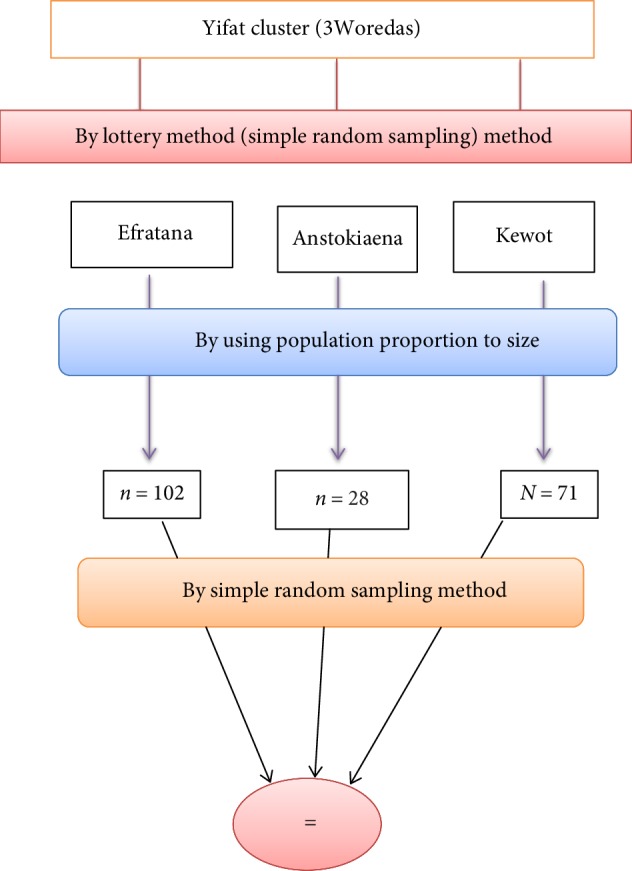
Schematic representation of sampling procedure on implementation of IMNCI among health care professional in North Shewa zone, Ethiopia, 2018.

The study done in Kenya categorized the factors affecting IMNCI implementations at three levels which include the challenges at Health worker level, facility level and community level. The health workers perception on skills uptake and case management guidelines is the main challenges at health worker level while time constraints (time taken to complete protocol, long queues, short staffing) and inadequate facility support (medical equipment, job aids, drugs) are identified as challenges at facility level. At community level long wait times, high cost of user fees and non-compliance by care giver and patients are identified as the main challenges to implement IMNCI guidelines [60]. Broader health systems factors appear to challenge IMNCI implementation are revealed by study conducted in Tanzania as constraints to the expansion of IMNCI training coverage (costs and high turnover of trained worker), factors underlying poor compliance (low training coverage, lack of health worker compliance, health system weakness), health system constraints to health worker compliance (lack of supplies, health facility set up, and human resource shortage) and limited means to document case management process which include limited supply of IMNCI materials for staff in facilities, lack of recording forms [19]. The study conducted in Botswana and Tanzania found several categories of factors affecting implementation of IMNCI with their subcategories 57% of participants confirmed as the procedure is too long and 45% report time consuming, 72% spent 10–29 minutes on one case, 5% reported physical layout is not good for procedure and negative attitude from supervisors 42% and lack of supportive supervision for the past six month 100%, lack of follow up training 78%, lack of uniformity between IMNCI trained and untrained nurse 32%, inadequate pre service training only 21%, due to cost of training 64% attended five day training despite recommended day is 11, 56.3% uses guideline only when there were few patient to attend, inadequate jobs aid for follow up 27.6% lack of knowledge were reported [61, 62]. In general different studies have been done worldwide on the factors influencing the implementation of the IMNCI strategy with different recommendations, but still there are gaps that shows as the implementation of the strategy is still poor especial in low resource setting countries like Ethiopia (Figures 1 and 2).

### 2.7. Conceptual Framework

## 3. Objectives

### 3.1. General Objective

To assess factors affecting the implementation of integrated management of neonatal and childhood illness for treatment of under five children by health professional in health care facilities in Yifat cluster in North Shewa Zone, Amhara region, Ethiopia, 2018.

### 3.2. Specific Objectives

- To assess the implementation of IMNCI by health professional in health care facilities.
- To identify factors affecting the implementation of IMNCI by health professional in health care facilities.

## 4. Methods and Materials

### 4.1. Study Area and Period

The study will be conducted in Yifat cluster which is located 190 km from Addis Ababa. The total population of Yafa clusteris = 323571. This cluster has three Woredas (Efereata Gedem = 170425, Antsokia Gemza = 62475 and Kewet = 90671). There are 360 health professionals in this cluster. This study will be conducted from March, 2018 to May 2018.

### 4.2. Study Design

Institutional based cross sectional study design will be conducted.

### 4.3. Populations

#### 4.3.1. Source of Population

Source population will be included all health care professionals in gov'tal health facilities in Yafat cluster.

#### 4.3.2. Study Population

All health professionals who are working in selected health facilities and present on days of data collection.

### 4.4. Eligibility Criteria

#### 4.4.1. Inclusion Criteria

Health professionals who are working in selected health facilities and present on days of data collection.

#### 4.4.2. Exclusion Criteria

Those health professionals who are sick, maternity and annual leave and also not available on dates of data collection.

### 4.5. Sample Size Determination and Sampling Techniques

#### 4.5.1. Sample Size

Single population proportional formula will be used to determine sample size based on the following assumptions: 95% level of confidence (*Zα*/2 = 1.96), the proportion of the implementation IMNCI (*p* = 58.4% (63)) and margin of error (*d* = 5%). The sample size will be calculated as follow:

(1) $$\begin{array}{c} ni={\left(Z_{a/2}\right)}^{2}\;P\;\left(1-P\right)={\left(1.96\right)}^{2}\left(0.584\right)\left(\;0.416\right)=373. \end{array}$$

*n* = 373, 

where: *n* = sample size. The total number of health care provider is 360 in Yifat cluster. So since this figure is below 10,000, use the following adjustment formula for the sample size:

(2) $$\begin{array}{c} n=\frac{n}{\left(1+n/N\right)}. \end{array}$$

where, *n* = sample size for population of size above 10,000. *N* = number of health care providers in North Shewa zone, Therefore, *n* = 373/(1 + 373/360), *n* = 183. The sample size will be **201** by using 10% none response rate.

#### 4.5.2. Sampling Techniques

First, total sample size (201) will be estimated based on the total number of health professionals in the Yifat cluster. There are 3Woredas in this cluster and each Woreda has its own health professionals. Next the determined sample is proportionally allocated to each Woreda. Finally, Simple Random Sampling method will be used to select study units from professional's staff to be included in the study based on the number of professional of each Woreda in the each Woreda as sampling frame.

### 4.6. Variables

#### 4.6.1. Independent Variable

- Socio-demographic characteristics
- Year of service Training
- Duration of training
- Health system layout
- Job training
- Resources/supplies
- Unique features of the IMNCI strategy
- Attitude of the health care professional

#### 4.6.2. Dependent Variable

- IMNCI implementation

### 4.7. Operational Definitions

#### 4.7.1. IMNCI Implementation

Is an application of the strategy or the guidelines in a comprehensive and holistic manner [11].

#### 4.7.2. Comprehensive Implementation

Ability of the facility to equip complete essential drugs & equipment for the five conditions (Malaria, Pneumonia, Diarrhea, Measles and Malnutrition) affecting children below five years of age.

#### 4.7.3. Holistic Intervention

Health managing of children below five years from overlapping disease symptoms and signs, by critically assessing children before prescribing a comprehensive and holistic therapy for Malaria, Pneumonia, Diarrhea, and Measles, Malnutrition at ago. The level of implementation can be categorized as high level and low level [11].

#### 4.7.4. High Level Implementation

Comprehensive implementation of IMNCI strategy above 68%.

#### 4.7.5. Low Level Implementation

Compressive implementation of IMNCI strategy below 68% [11].

### 4.8. Data Collection Tool and Procedure

Structured self-administered questionnaires will be used which is adopted from literatures [52, 53]. After 4 data collectors (diploma nurses) will be employed, training will be given for one day on clarification of some terms and assessment tools.

### 4.9. Data Quality Management

To assure high quality of data, training will be given for data collectors and the questionnaire will be pre-tested on 5% health care professional in other health care facility before the main survey with modification Keyit health center. The collected data will be reviewed and checked for completeness before data entry; the incomplete data will be discarded.

### 4.10. Data Collection and Analysis

Data will be collected by using, structured self-administered questionnaires [52, 53]. For data analysis SPSS version 20 will be used. Univariate (frequency), Bivariate, Multiple logistic regression analysis will be employed. *P*-value and 95% confidence interval (CI) for OR will be used in judging the significance of the associations. *P*-value less than 0.05 will be taken as significant association.

### 4.11. Ethical Consideration

Ethical clearance was obtained from Debre Birhan health College science. Informed consent will be obtained from a respondent who will be participated in the study. Confidentiality will be maintained by omitting their name and personal identification and participant will not be compelled to the study.

### 4.12. Dissemination of the Result Plan

The results of this study will be disseminated to Debre Birhan health College science, North Shewa zone health office, Woredas health bureau, and other concerned bodies through reports and publication on an appropriate journal.

## 5. Results

### 5.1. Socio-Demographic Characteristics of Study Population

A total of 201 health care professionals were involved making the response rate 100%. Among the study population 142 (70.6%) were males. Regarding the age of the respondents the more than half 112 (55.7%) was aged between 25 and 29 years. Concerning religion, 115 (57.2%) of the health care professionals are Muslim. With regard to level of qualification 116 (58.2%) were diploma health care professionals while 80 (39.8%) were BSc health care professionals. Among the study participants 112 (55.7%) were currently married and the majority, 177 (88%) were belong to Oromo ethnic group (Table 1).

### 5.2. Training Related and Factors Affecting the Implementation of the IMNCI Strategy

More than half, 119 (59.2%) of respondents are served as health care provider for less than five years. Of the total, 162 (80.6%) respondents worked in under five outpatient department. One hundred fifty nine (79%) of the participants are served in under five OPD between 0 and 5 years.

Regarding IMNCI training, the nearly half 116 (57.7%) of respondents attended IMNCI training at different periods, at which the less than half 68 (33.8%) of participants attended between 2005 and 2009 years. Of the total, who had attended training the almost half 103 (51.24%) of respondents attended in service training. One hundred seventy nine (89.1%) of study participants are not received follow up training.

Concerning the factor influencing the implementation of IMNCI strategy, more than half, 113 (56.2%) reported that lack of trained staff and lack of supplies, 75 (37.3%) as the main challenges of IMNCI implementation (Table 2).

### 5.3. Duration of IMNCI Training

Almost half off the study participants, 55 (51.7%) took their IMNCI training for six days and followed by those who underwent their training for eight days 21 (11.4%) (Table 3).

### 5.4. Completion of IMCI-1 Training as a Criterion in the Assignment of Daily Duties to Health Care Professionals

The majority, 177 (75.1%) of study participants are reported that completion of IMCI training is considered in the assignment of daily duties to nurses at their respective facilities (Table 4).

### 5.5. Steps in the Case Management Protocol that were Found Difficult to Apply

The more than half of the study participants reported that all steps in the IMCI case management protocol were always found difficult for them to apply. Out of the six steps of IMNCI case management protocol the nearly half, 111 (55.2%), 104 (51.7%), 101 (50.2%) found the steps provide follow up, Identify the treatment and classify the child illness were most difficult steps, respectively (Table 5).

### 5.6. IMNCI Activities Performance of the Study Participants

The majority of the study participants, 180 (89.6%), 182 (90.5%), 184 (91.5%), 179 (89%), 179 (89%), 156 (77.6%), 174 (86.6%), 165 (82.1%), 143 (71.1%), 172 (85.6%) were always performing checking for vaccination, danger sign, pallor, assessing fever, Diarrhea, malaria, cough, weighing children, checking weight against chart and checking for ear problem respectively (Table 6).

### 5.7. Application of the Steps in the Case Management Protocol

One hundred thirty five 135 (67.1%) of the study participants always apply all stages of integrated case management protocol followed those who are applying most of the stages of integrated case management protocol 56 (27.9 %) (Table 7).

### 5.8. The Effect of Using the IMCI Protocol on the Patient Health Care Providers' Ratio

According to this study, 112 (55.7%) strongly agreed and 80 (39.8%) agreed indicated that they provide health education to the caregivers of every child that they manage using the IMNCI protocol.

With regard to the effect of using the IMNCI protocol on the patient-health care professional ratio, 43 (21.4%) strongly agreed and agreed 92 (45.8%) of respondents stated that if they apply all the steps in the IMNCI case management protocol to every under-5 patient that they tend, they will only be able to see a handful of the under-5 patients.

Regarding the general patient-nurse ratio, 94 (23 strongly agreed and 71 agreed) (46.7%) of the respondents disclosed that if they apply all the stages of the IMNCI case management protocol to all under-5 patients, they would not be able to attend to all the other patients who are not under-5s, because of the low general patient-health care professionals ratio (Table 8).

### 5.9. Time Spent Managing an Under-5 Patient When Using the IMCI Case Management Protocol

According to this study, 9 (4.4%) and 27 (13.4%) of respondents were strongly agreed and agreed that they spent more than one hour when using IMNCI protocol whereas 11 (5.5%) and 25 (12.4%) of study participants were strongly agreed and disagreed on spending between 30 and 40 min when not using IMNCI protocol.

Based on this study, 16 (8%) and 97 (48.2%) of respondents were strongly agreed and agreed on spending of time between 30 and 40 minutes when using IMNCI protocol which is above WHO recommended consultation (15–20 minutes) while, 17 (8.5%) and 94 (46.8%) of respondents were strongly agreed and agreed on spending between 30 and 40 minutes when not using IMNCI protocol.

Similarly, 52 (25.9%) and 97 (48.2%) of respondents were strongly agreed and agreed on spending of time between 10 and 29 minutes when using IMNCI protocol whereas 43 (21.4%) and 90 (44.8%) of respondents were strongly agreed and agreed on spending between 10 and 29 minutes when not using IMNCI protocol.

The time estimate of 1–9 minutes attracted differing responses from the study participants. Only 51 (25.4%) participants explained that they spend 1–9 minutes with an under-5 patient when using the IMCI protocol, while 67 (33.4%) of study participants stated that they spent 1–9 minutes with an under-5 patient when not using the IMCI protocol (Tables 9 and 10).

### 5.10. The Impact of IMCI on Case Management Skills

According to this study, 94 (46.8%) and 49 (24.4%) of study participants were strongly agreed and agreed respectively that IMNCI training had boosted their self-confidence and skills in managing patients under 5 years of age.

Regarding the question about waiting queues as a result of the amount of time spent applying all the steps in the IMNCI protocol;36 (17.9%)% and 84 (41.8%) of study participants were strongly agreed and agreed, respectively mentioned that IMCI had led to longer waiting queues.

Concerning the perception that IMCI is partially implemented because non-IMNCI-trained health care professionals take over the care of other children waiting in queues if it is felt that the IMNCI-trained health care professionals are taking too long to assess a patient mixed responses were seen. Related this, 30 (14.9%) and 72 (35.8%) of respondents were strongly agreed and agreed, respectively that IMNCI was being partially implemented in some health facilities within the zone.

With regard to whether IMNCI had reduced the number of follow-up visits by under-5 patients, 81 (40.3%) agreed and 38 (18.9%) strongly agreed that IMNCI had reduced the number of follow up visits by under-5 patients.

Concerning about the feasibility of always referring to the IMNCI chart booklet during the case management of under-5 patients. Ninety five (47.2%) indicated that it is always possible to refer chart booklet during case management of every presentation, while 49 (24.4%) and 35 (17.4%) of study participants were strongly agreed and disagreed in their response respectively.

Regarding about inconsistency of case management practices of IMNCI trained and non-IMNCI trained health care professionals, 85 (42.3%) and 37 (18.4%) of participants were agreed and strongly agreed in their response respectively on which under-5 patients should be seen by IMNCI trained health care professionals (Table 11).

### 5.11. Experiences of the Health Care Providers in Implementing the Guidelines and Procedures of the IMCI Strategy

According this study, 99 (50.3%) and 80 (41.6%) strongly agreed and agreed with the perception that the IMCI strategy is user-friendly to health workers. Regarding the idea whether IMNCI is easy to understand and apply, 76 (37.8%) and 87 (43.2%) of respondents were strongly agreed and agreed respectively in which IMNCI is easy to understand and apply. Out of the total respondents, 80 (39.8%), 84 (41.8%), 69 (34.4%), and 147 (73.1%) disagreed with notion that IMNCI protocol is too long, IMNCI protocol is tedious, IMNCI is not practical at our health facility and IMNCI is time consuming, respectively (Table 12).

### 5.12. Supervision

The majority of the respondents, 87 (43.3%) disagreed and 28 (13.9%) strongly disagreed with notion my supervisor does not appreciate the rationale for IMNCI. Similarly, 95 (47.3%) and 45 (22.4%) of respondents disagreed and strongly disagreed with the statement my supervisor is not IMNCI trained. About lack of supervision by IMNCI trainers. Among the study participants 80 (39.8%) agreed with the statement while 60 (29.9%) respondents disagreed with the statement lack of supervision by IMNCI trainers (Table 13).

### 5.13. Availability of Resources to the IMNCI Strategy

Regarding availability of resources 87 (27 = strongly agree and 60 = agree) (43.3%) of respondents agreed that IMNCI drugs are frequently out of stock while 111 (96 = disagree and 15 = strongly disagree) (55.3%) of respondents disagreed with the statement IMNCI wall charts and chart booklets are frequently unavailable. Concerning about health facility equipment support, 95 (strongly agree = 26 and agree = 69) (47.2%) agreed that their health facility is not fully equipped to support the use of IMNCI strategy (Table 14).

### 5.14. Attitudes of Health Care Professionals towards IMCI

Regarding some health care professionals attitude toward the strategy, 100 (77 = disagree and 23 = strongly disagree) participants (49.7%) disagreed with the notion some health care professionals have negative attitude towards IMNCI strategy Based on this study, 74 (strongly agree = 18 and agree = 56) study participants (36.9%) agreed that IMNCI guild lines are too simplistic to give care for under five children (Table 15).

### 5.15. Training of Implementers

Concerning about lack of follow up training by IMNCI facilitators, most of the respondents 141 (22.9% = strongly agreed and 47.3% = agreed) revealed that lack of IMNCI follow up training by IMNCI facilitators are the main challenges of implementing strategy (See Table 16).

### 5.16. Association of Factors Influencing IMNCI Implementation by Health Care Professional in Yifat Cluster of North Shewa Zone

In bivariate analysis, Attended IMNCI training, Lack of supervision, IMNCI is difficult to understand and apply, supervisor does not appreciate rationale for IMNCI, always apply of all stages of IMNCI and always referring chart booklet had significant association the dependent variable. By using multivariate logistic regressions analysis the above listed variables were adjusted. Among those variables only two variables were significantly associated with the implementation of IMNCI strategy by health care professionals in public health institutions in Yifat cluster.

Training and always referring chart booklets was independent predictor of IMNCI implementations. Those health care providers who had attended training were 2.6 times more likely to implement IMNCI than those who had not attended IMNCI training [*AOR* = 2.7, 95% CI: (1.1.278, 4.562)].Similarly, those health care professionals who had the practice of always referring IMNCI chart booklet during every presentation of case management process were 2.76 times more like to implement IMNCI protocol than those who do not always refer chart booklet [*AOR* = 2.76, 95% CI: (1.753,5.975)] (Table 17).

## 6. Discussion

This study aimed to identify the IMNCI implementation and factors influencing IMNCI implementation by nurses in public health institutions of Yifat cluster in North Shewa zone. The study finding revealed that proportion of IMNCI implementation was 58% high level implementation. This finding was similar with the study done in West Arsi zone in Ethiopia (63). This is below standard level established (above 68%), by WHO and UNICEF due to presence so many challenges, however, this finding was quite higher than that of the study conducted in Kenya and China which showed that, utilization and application of IMNCI concepts in the region is approximately 14% [13]. This study also contrary from the study conducted in Benin which showed Performance of individual health workers varied greatly, from 15% to 88% of patients treated correctly, on average in accordance with the IMNCI guidelines [10, 11].

Based on this study, 57.7% of study participants had attended IMNCI training which is still below WHO recommendation that at least 60% of health care workers seeing sick children in the health facilities are trained in IMNCI. This finding was in line with the done in Arsi zone (63). However, the present finding is higher than that of the study conducted in Indonesia which showed only 43% IMNCI trained health workers. This slight difference might be due trained staff turnover and high cost of training [46, 47].

According this study, the health care providers who had attended training were 2.6 times more likely to implement IMNCI than those who had not attended IMNCI training [*AOR* = 2.7, 95% CI: (1.1.278, 4.562)].This finding was in line with in study done in Arsi zone (63). Similarly , health care professionals who had the practice of always referring IMNCI chart booklet during every presentation of case management process were 2.76 times more like to implement IMNCI protocol than those who do not always refer chart booklet [*AOR* = 2.76, 95% CI: (1.753, 5.975)]. This finding is almost consistent with study conducted in Arsi zone (63).

In this study, correctly classify illness, prescribe medication, and treat the children were not significantly associated with the dependent variable. Opposite to this finding, in systematic review and meta study conducted in lower resource setting country indicated that IMNCI trained workers were two times more likely to correctly classify illness and three times more likely to prescribe medication and treat the children [20]. Similarly another study also revealed that IMNCI trained health workers are significantly more likely prescribing correct treatment than no IMNCI trained workers [14].

Based on this study, only 10.9% of participants received follow up training. This study finding was consistent with the study done in Arsi zone in Ethiopia (63). The present finding is lower than the figures (78%) reported in studies conducted in Botswana and Tanzania [61, 62].

Broader health systems factors appear to challenge IMNCI implementation [19]. In present study several factors were identified as hindrance to IMNCI implementation. Some of the factors include untrained staff (56.2%), lack of supervision (27.4%), lack of supplies (37.3%), lack of good attitude (11.9%) and shortage of staff (16.4%) were the major identified factors. Similarly, lack of supplies (37.3%), frequent unavailability of IMNCI drugs (43.78%) and wall charts and chart booklets (39.4%) and unequipped health facility (49.7%) were the major identified factors in Arsi study (63). Inconsistent to this study the study conducted in Botswana showed that only 10% of respondents agreed that IMNCI recommended drugs are often out of stock and 15% of the respondents claimed that IMCI chart booklets and wall charts are often unavailable in their health facilities while Thirty-six percent of the participants responded that their health facilities are not fully equipped to support the application of IMCI skills and procedures. This difference might be due to difference in socio economic status the study area and sample sizes. In another way several studies done in different parties of the world identified similar challenges of IMNCI implementations [50–52, 55–57, 60].

Concerning health workers related factors, in this study many factors were identified as the challenge to IMNCI implementations. Among these factors, shortage of staff (16.4%), lack of good knowledge (8.5%), untrained staff (56.2%) and uncovered patient — health care workers ratio (32.8%) were identified as the hinder of IMNCI implementation by health care workers in public health institution. This study was inline the study done in Arsi zone in Ethiopia (63). This was inconsistent with other studies, for instance study conducted in Indonesia and Tanzania revealed that shortage of trained staff were 43% and 49%respectively [46, 47]. This difference might be due to difference in economic status, study area and study period. In another way this finding is consistent with other studies in Africa [17, 19, 50–57, 60, 62].

Regarding some health care professionals attitude toward the strategy, 100 participants (49.7%) disagreed with the notion some health care professionals have negative attitude towards IMNCI strategy and 74 study participants (36.9%) agreed that IMNCI guild lines are too simplistic to give care for under five children. Similarly, some clinician‘s and doctors are towards IMNCI was also identified in other health workers in Kenya and south Africa in which some doctors and clinical officers failed to accept the IMNCI approach, and they also felt that the guidelines are too simplistic and do not allow for full use of their clinical training [60].

This study also tried to identify about IMNCI feature related barriers of IMNCI implementation by health care workers like the IMNCI protocol is too long (59.7%), IMNCI protocol is tedious (41.3%), and IMNCI is time consuming (55.2%),and IMNCI easy not to understand and apply (11%).This finding was almost in line with the study done in Arsi zone (63).In the Tanzanian CREHS policy documented that, the IMCI protocol is perceived as unnecessarily time consuming, and that, health workers sometimes cut corners so that they can attend to other clients awaiting their attention [19, 49]. An average consultation of this study was also supported with WHO recorded that —an average-16 minutes, IMCI about 2–4 minutes consultation longer than traditional consultation. Similarly another several studies also listed this factors as the barrier implement IMNCI protocol [55, 56, 60].

### 6.1. Limitation

It was cross sectional study that did not show cause and result relation (It has chicken eggs dilemma).

The result of this study depended on self-report of health care professionals and as a result there might be influence of social desirability; nevertheless, the study was confidential and data collectors were instructed to guarantee that their responses could not be related to them.

Our study was limited to Yifat cluster of North Shewa zone and hence the findings cannot be generalized to the Amhara region or the country as a whole.

## 7. Conclusion

This study revealed that the proportion of IMNCI implementation in the study setting was low (58%).

Only 57.7% of Health care workers/professionals have reported to attend IMNCI training as compared to WHO recommendation (60%).

This study identified barriers by health care workers which include: shortage of essential drugs and supplies, inadequate trained staff, time consuming nature of the protocol, lack of supervision, lack of knowledge about the strategy and lack of good attitude of healthcare workers/professionals towards the IMNCI strategy.

## 8. Recommendation

- As IMNCI training is an important predictor of IMNCI implementation, there should be increasing the number of health workers trained in IMNCI and capacitated to deliver IMNCI services by Scaling up both pre-service and in service IMNCI training. Therefore, policy makers and concerned stakeholders, government and non-government bodies should give great emphasis to training.
- Ministry of health, Regional health bureau, zonal health bureau, NGOs and other concerned bodies should have work together to provide a facility layout that allows for space and time to apply IMNCI skills and procedures.
- Lack of supplies like shortage essential IMNCI drugs and chart booklet should be minimized by involving NGOs such as Save the children and other concerned bodies.
- IMCI facilitators should give emphasis on IMNCI follow-up training and visits to IMNCI implementers.
- Training of senior manager in IMNCI strategy to boost their confidence in supervising IMNCI protocol implementers.
- Health center manager need to consider IMNCI training during daily assignment of health care workers in under five OPD.
- Further Large scale study with a representative sample size is recommended to be conducted in the future.

## Figures and Tables

**Table 1:** Socio-demographic characteristics of respondents among health care professionals in Public health institution of Yifat, North Shewa zone April, 2018 (*n* = 201).

Variables	Category	Frequency	Percent
Sex	Male	142	70.6
	Female	59	29.4

Age of respondents	20–24	62	30.8
	25–29	112	55.7
	30–34	10	4.9
	35–39	17	8.5

Level of qualification	MSc	4	2
	BSc	80	39.8
	Diploma	116	58.2

Marital status	Married	112	55.7
	Single	86	42.8
	Divorced	3	1.5

Religion	Orthodox	48	23.9
	Muslim	115	57.2
	Protestant	38	18.9

Ethnicity	Oromo	177	88
	Amhara	22	12

**Table 2:** Distribution of Training related and factors affecting the implementation of the IMNCI strategy among health care professionals in public health institution of Yifat cluster, North Shewa zone April, 2016. (*n* = 201).

Variable	Frequency	Percent
*Year of service as healthcare provider*		
<5 years	**119**	59.2
6–10 years	**67**	33.3
>10 years	15	7.5

*Worked in under five OPD*		
Yes	162	80.6
No	39	19.4

*Year of service in under five OPD*		
0–5 years	159	79
>=6 years	3	1.5

*Attended IMNCI training*		
Yes	116	57.7
No	85	42.3

*Types of IMNCI training*		
Pre service	13	6.5
In service	103	51.24

*Period of last IMNCI training*		
2001–2004	28	13.9
2005–2009	68	33.8
2010–2014	2	1
2015^+^	17	8.5

*Received follow up training*		
Yes	22	10.9
No	179	89.1

*Period of IMNCI follow up training*		
2000–2004	1	0.5
2005–2009	19	9.5
2010–2014	3	1.5
2015^+^	6	3

*Factors influencing the implementation of IMNCI Strategy*		
Shortage of staff	33	16.4
Time consuming	66	33
Lack of supplies	75	37.3
Untrained staff	113	56.2
Lack of supervision	55	27.4
Lack of knowledge	17	8.5
Lack of good attitude	24	11.9

**Table 3:** Duration of IMNCI training.

Duration of training	Frequency	Percent
5 days	12	5.9
6 days	104	51.7
7 days	30	14.9
8 days	40	19.9
9 days	7	3.5
10 days	8	4

**Table 4:** IMNCI training considered during daily duties.

Variable	Category	Frequency	Percent
IMNCI training considered during daily duties	Yes	177	75.1
	No	48	24.9

**Table 5:** Steps in the case management protocol that were found difficult to apply.

Variables	Category	Frequency	Percent
Provide follow up	Always	111	55.2
	Some times	40	19.9
	Not Difficult	50	24.9

Counsel the caretaker	Always	95	47.3
	Some times	32	15.9
	Not difficult	74	36.8

Treat the child	Not difficult	68	33.8
	Some times	34	16.9
	Always	97	48.3

Identify treatment	Not difficult	77	38.3
	Some times	20	10
	Always	104	51.7

Classify the child illness	Not difficult	64	31.8
	Some times	36	18
	Always	101	50.2

Assess the child	Not difficult	56	27.9
	Some times	48	23.9
	Always	97	48.2

**Table 6:** IMNCI activities performance of the study participants.

Variables	Category	Frequency	Percent
Checking for vaccination	Always	180	89.6
	Sometimes	17	8.5
	Not performed	4	1.9

Checking for danger signs	Always	182	90.5
	Sometimes	16	8
	Not performed	3	1.5

Checking for pallor	Always	184	91.5
	Sometimes	13	6.5
	Not performed	4	2

Assessing fever	Always	179	89
	Sometimes	17	8.5
	Not performed	5	2.5

Assessing diarrhea	Always	179	89
	Sometimes	17	8.5
	Not performed	5	2.5

Assessing malaria	Always	156	77.6
	Sometimes	43	21.4
	Not performed	2	1

Assessing cough	Always	174	86.6
	Sometimes	24	11.9
	Not performed	3	1.5

Weigh the children	Always	165	82.1
	Sometimes	33	16.4
	Not performed	3	1.5

Check wt against chart	Always	143	71.1
	Sometimes	52	25.9
	Not performed	6	3

Checking for ear problem	Always	172	85.6
	Sometimes	26	12.9
	Not performed	3	1.5

**Table 7:** Application of the steps in the case management protocol.

Variable	Category	Frequency	Percent
Application of the steps in the IMNCI	Always apply all stages of IMNCI	135	67.1
	Apply most stages of IMNCI	56	27.9
	Do not apply any stages of IMNCI	10	5

**Table 8:** The effect of using the IMCI protocol on the patient health care professionals' ratio.

Variables	Category	Frequency	Percent
Provide health education to care taker	Strong agree	112	55.7
	Agree	80	39.8
	Neutral	5	2.5
	Disagree	2	1
	Strong disagree	2	1

Under 5 patient health care professional ratio	Strong agree	43	21.4
	Agree	92	45.8
	Neutral	21	10.4
	Disagree	27	13.4
	Strong disagree	18	9

General patient — health care professional ratio	Strong agree	23	11.4
	Agree	71	35.3
	Neutral	12	6
	Disagree	80	39.8
	Strong disagree	15	7.5

**Table 9:** Time spent managing an under-5 patient when using the IMCI case management protocol.

When using IMNCI protocol	Response	Frequency	Percent
More than one hour	Strong agree	9	4.4
	Agree	27	13.4
	Neutral	14	7
	Disagree	118	58.7
	Strong disagree	33	16.4

30–40 minutes	Strong agree	16	8
	Agree	97	48.2
	Neutral	21	10.4
	Disagree	59	29.4
	Strong disagree	8	4

10–29 minutes	Strong agree	52	25.9
	Agree	97	48.2
	Neutral	19	9.5
	Disagree	30	14.9
	Strong disagree	3	1.5

1–9 minutes	Strong agree	19	9.5
	Agree	32	15.9
	Neutral	21	10.4
	Disagree	96	47.8
	Strong disagree	33	16.4

**Table 10:** Time spent managing an under-5 patient when not using the IMCI case management protocol.

When do not using IMNCI protocol	Response	Frequency	Percent
More than one hour	Strong agree	11	5.5
	Agree	25	12.4
	Neutral	16	8
	Disagree	103	51.2
	Strong disagree	46	22.9

30–40 minutes	Strong agree	17	8.5
	Agree	94	46.8
	Neutral	20	10
	Disagree	60	29.9
	Strong disagree	10	5.8

10–29 minutes	Strong agree	43	21.4
	Agree	90	44.8
	Neutral	9	4.4
	Disagree	54	26.9
	Strong disagree	5	2.5

1–9 minutes	Strong agree	17	8.5
	Agree	50	24.9
	Neutral	21	10.4
	Disagree	83	41.3
	Strong disagree	30	14.9

**Table 11:** The impact of IMCI on case management skills.

Variables	Response	Frequency	Percent
IMNCI boosted continue	Strong agree	94	46.8
	Agree	49	24.4
	Neutral	22	10.9
	Disagree	25	12.4
	Strong disagree	11	5.5

IMNI led to long waiting time	Strong agree	36	17.9
	Agree	84	41.8
	Neutral	30	14.9
	Disagree	40	19.9
	Strong disagree	11	5.5

IMNCI is partial implemented	Strong agree	30	14.9
	Agree	72	35.8
	Neutral	28	13.9
	Disagree	58	28.9
	Strong disagree	13	6.5

IMNCI has reduced number of follow up visits	Strong agree	38	18.9
	Agree	81	40.3
	Neutral	16	8
	Disagree	38	18.9
	Strong disagree	28	13.9

Not practical to refer IMNCI always	Strong agree	27	13.4
	Agree	68	33.8
	Neutral	22	10.9
	Disagree	49	24.4
	Strong disagree	35	17.4

All IMNCI trained health care professional	Strong agree	37	18.4
	Agree	85	42.3
	Neutral	23	11.4
	Disagree	45	22.4
	Strong disagree	11	5.5

**Table 12:** Experiences of the health care providers in implementing the guidelines and procedures of the IMCI strategy.

Variables	Category	Frequency	Percent
IMNCI is a user friendly strategy	Strong agree	99	49.2
	Agree	80	39.8
	Neutral	7	3.5
	Disagree	11	5.5
	Strong disagree	4	2

IMNCI easy to understand and apply	Strong agree	76	37.8
	Agree	87	43.2
	Neutral	16	8
	Disagree	16	8
	Strong disagree	6	3

	Strong agree	16	8
	Agree	83	41.3
	Neutral	22	10.9
	Disagree	76	37.8
	Strong disagree	4	2

IMNCI protocol is tedious	Strong agree	17	8.5
	Agree	66	32.8
	Neutral	34	16.9
	Disagree	75	37.3
	Strong disagree	9	4.5

IMNCI is time consuming	Strong agree	27	13.4
	Agree	84	41.8
	Neutral	21	10.4
	Disagree	54	26.9
	Strong disagree	15	7.5

IMNCI is not practical at our health institution	Strong agree	16	8
	Agree	23	11.4
	Neutral	15	7.5
	Disagree	109	54.2
	Strong disagree	38	18.9

**Table 13:** Supervision.

Variables	Response	Frequency	Percent
Supervisor doesn't appreciate rational for IMNCI	Strong agree	11	5.5
	Agree	43	21.4
	Neutral	32	15.9
	Disagree	87	43.3
	Strong disagree	28	13.9

Supervisor not IMNCI trained	Strong agree	24	11.9
	Agree	26	12.9
	Neutral	11	5.5
	Disagree	95	47.3
	Strong disagree	45	22.4

Lack of supervision by IMNCI trainers	Strong agree	40	19.9
	Agree	80	39.8
	Neutral	10	4.9
	Disagree	60	29.9
	Strong disagree	11	5.5

**Table 14:** Availability of resources to the IMNCI strategy.

Variables	Response	Frequency	Percent
IMNCI drugs are frequently out of stock	Strong agree	27	13.4
	Agree	60	29.9
	Neutral	14	7
	Disagree	78	38.8
	Strong disagree	22	10.9

IMNCI wall charts and chart blooket are frequently unavailable	Strong agree	20	10
	Agree	59	29.3
	Neutral	11	5.4
	Disagree	96	47.8
	Strong disagree	15	7.5

Health facility is not fully equipped to support the use of IMNCI	Strong agree	26	12.9
	Agree	69	34.3
	Neutral	16	8
	Disagree	84	41.8
	Strong disagree	6	3

**Table 15:** Attitudes of health care professionals towards IMCI.

Variables	Response	Frequency	Percent
Some health care professional have negative attitude towards IMNCI	Strong agree	18	9
	Agree	56	27.9
	Neutral	27	13.4
	Disagree	77	38.3
	Strong disagree	23	11.4

IMNCI guidelines too simplistic it undermines clinical training	Strong agree	27	13.4
	Agree	79	39.3
	Neutral	10	5
	Disagree	76	37.8
	Strong disagree	9	4.5

**Table 16:** Training of implementers.

Variable	Response	Frequency	Percent
Lack of IMNCI follow up training	Strong agree	46	22.9
	Agree	95	47.3
	Neutral	9	4.5
	Disagree	40	19.9
	Strong disagree	11	5.4

**Table 17:** Association of factor influencing IMNCI implementation by health care professionals adjusted to confounding variables in Yifat cluster in North Shewa zone, April 2016 (*n* = 201).

Variable	IMNCI implementation		COR (95% CI)	AOR (95% CI)	*P*-value
	High level	Low level			
*Attended IMNCI training*					
Yes	80 (39.8%)	36 (17.9%)	**2.88 (1.371,5.273)**	**2.6 (1.278,4.562)**	**0.002**
No	37 (18.4%)	48 (23.9%)	1	1	

*Lack of follow up training*					
Yes	74 (36.8%)	65 (32.3%)	0.5 (0.123,2.182)	1.2 (0.135,1.446)	0.065
No	43 (21.4%)	19 (9.5%)	1	1	

*Lack of supervision*					
Yes	62 (30.8%)	58 (28.9%)	1	1	0.015
No	55 (27.4%)	26 (12.9%)	**1.97 (1.083,4.569)**	2.15 (0.768,1.575)	

*IMNCI is difficult to understand and apply*					
Yes	14 (7%)	23 (11.4%)	1	1	0.067
No	103 (51.2%)	61 (30.4%)	**2.77 (1.382,4.945)**	1.6 (1.467,2.758)	

*Supervisor does not appreciate rationale for IMNCI*					
Yes	23 (11.4%)	26 (12.9%)	1	1	0.084
No	95 (47.3%)	57 (28.4%)	**1.88 (2.021,5.677)**	3.4 (2.147,6.812)	

*Always apply of all stages of IMNCI*					
Yes	89 (44.3%)	47 (23.4%)	**2.502 (1.44, 5.76)**	1.3 (0.821, 5.392)	0.0237
No	28 (13.9%)	37 (18.4%)	1		

*Always referring chart booklet*					
Yes	67 (33.3%)	33 (16.4%)	1	1	**0.01**
No	5024.9%)	51 (25.4%)	**2.07 (1.53, 4.732)**	**2.76 (1.753, 5.975)**	

Bold values show a significant association of independent variables with dependent variables.

## Data Availability

Data supporting the conclusions of this article are available by request to Ayele Mamo Abebe. The relevant raw data will be made available to researchers wishing to use them for noncommercial purposes. The Supplementary data will be put below the acknowledgement.

## Abbreviations

AIDS: Acquired immunodeficiency syndrome
CAH: Child and adolescent health and development
EDHS: Ethiopian demographic health survey
FMOH: Federal minister of health
HEWs: Health extension workers
HC: Health center
HIV: Human immune virus
ICCM: Integrated community case management
IMCI: Integrated management of childhood illness
IMNCI: Integrated management of neonatal and child hood illness
MDG: Millennium developmental goal
PHCNs: Primary health care nurses
SDGs: Sustainable developmental goals
SNNPR: Southern nation, nationalities and people region
UNICEF: United nation international children emergency fund
UNIGME: United nations intergroup for mortality estimation
WHO: World health organization.

## Supplementary Materials

Supplementary Material Questionnaire Part 1: Socio-Demographic related Questionnaires nurses. Part 2. Questions related training and factors affecting the implementation the IMCI strategy. Part.3. Question related to nurses' experience in implementing the IMCI strategy.
