# Supplementary material for: Assessment of Factors Affecting the Implementation of Integrated Management of Neonatal and Childhood Illness for Treatment of under Five Children by Health Professional in Health Care Facilities in Yifat Cluster in North Shewa Zone, Amhara Region, Ethiopia
Source: Int J Pediatr. 2019 Dec 14;2019:9474612. doi: 10.1155/2019/9474612 (PMC6948312; doi:10.1155/2019/9474612)
Supplement: Supplementary Material — Questionnaire Part 1: Socio-Demographic related Questionnaires nurses. Part 2. Questions related training and factors affecting the implementation the IMCI strategy. Part.3. Question related to nurses' experience in implementing the IMCI strategy. [file 9474612.f1.pdf]

QUESTIONNAIRE

PART 1: Socio-Demographic related Questionnaires nurses.

All questionnaires are completed anonymously. We would appreciate if you answer all the questions and answer as honestly as possible. Please circle on the number you select that best answers the question. Kindly make only one selection unless otherwise instructed.

Ser.no	Socio-Demographic	Response	Remark
101	Sex	1. Male 2. Female	
102	Age	___in years	
103	Religion	1. Orthodox 2. Muslim 3. Protestant 4. Other(specify)_________	
104	level of qualification	1. MSc 2. BSc 3. Diploma 4. Other (specify) ____	
105	Marital status	1. Married 2. Single 3. Divorced 4. Widowed 5. Separated	
106	Ethnicity	1. Oromo 2. Amhara 3. Sidama<br>4. Other (specify)_____	

**Part 2. Questions related training and factors affecting the implementation the IMCI strategy.**			
201	How long have you been serving as health care provider?	1. <5 yrs. *2.* 5-10 yrs.<br>*3.* 10-15 yrs. *4.* >15yrs	
202	Have you ever worked in a Pediatric/Children ward or under five OPD?	1.Yes 2.No	
203	If yes to # 202, How long have you been serving in under five clinics?	1. 0-5 years 2. 6-10yrs<br>3. More than 10	
204	Have you attended IMNCI training?	1. Yes 2. No	
205	205 If yes to # 204, which IMCI training did you receive?	1.pre service training 2.in service	
206	When you last attended training (Year and	month_______ year________	

	month of training)?		
207	What was the duration of IMNCI training?	_____days	
208	Have you received IMCI follow-up training?	1. Yes 2. No	
209	If yes to # 208, When you last attended follow up training IMCI strategy?	Month ------Year______	
210	What are the main challenges encountered in implementing the IMCI strategy?	1. Shortage of staff 2. Time consuming 3. Lack of supplies/resource 4. Untrained staff 5. Lack of supervision<br>6. Lack of knowledge<br>7. Lack of good attitude 8.Others(specify)-----	

PART.3. Question related to nurses' experience in implementing the IMCI strategy

301. Is IMNCI training considered during the assignment of daily duties to nurses at this health facility? 1. Yes 2. No

302	How often the following case management steps or skills of the IMCI case management process do you find difficult to practice?			
		always	sometimes	Not difficult
302.1	Assess the child's condition			
302.2	Classify the child's illness			
302.3	Identify treatment			
302.4	Treat the child			
302.5	Counsel the caretaker			
302.6	Provide follow-up care			

303	How often do you perform the following IMNCI activities?			
303.1	Checking for vaccination			
303.2	Checking for danger signs			
303.3	Checking for pallor			
303.4	Assessing fever			
303.5	Assessing diarrhea			
303.6	Assessing malaria			
303.7	Assessing cough			
303.8	Weighing the children			
303.9	Checking weight against the chart			
303.10	303.10 Checking for ear problem			

**304**	Which of the following statements best describes your management of a sick child at your facility?					
304.1	always apply all the stages of the integrated case management process					
304.2	I apply most of the stages of the integrated case management process					
304.3	I do not apply any of the stages of the integrated case management process					
305	To what extent do you agree with the following statements?					
		Strongly agree	Agree	Neutral	Disagree	Strongly disagree
305.1	I provide health education to the parent/guardian/caretaker of every child I manage					

305.2	If I apply all the stages of the IMCI case Management process to all under 5 patients, I will be able to see only a handful of them because of the patient -nurse ratio.					
305.3	If I apply all the stages of the IMCI case management process to all under patients, I would not be able to attend to all other patients who are not under 5s due to the patient-nurse ratio					

**306**	Provide an estimate of time spent on the assessment and treatment of a child using the IMCIcase management protocol.					
		Strongly agree	Agree	Neutral	Disagree	Strongly disagree
306.1	I spend more than one hour per patient					
**306.2**	I spend 30-40 minutes per patient					
**306.3**	I spend 10-29 minutes per patient					
306.4	I spend 1-9 minutes per patient					
**307**	Provide an estimate of time spent on the assessment and treatment of a child without using the IMNCI case management protocol.					
**307.1**	I spend more than one hour per patient					
**307.2**	I spend 30-40 minutes per patient					
**307.3**	I spend 10-29 minutes per patient					

**307.4**	spend 1-9 minutes per patient					

308	To what extent do you agree with the following statements about IMCI strategy at your health facility?					
		Strongly agree	Agree	Neutral	Disagree	Strongly disagree
308.1	IMNCI has boosted my confidence and skills in Under 5 patient case management.					
308.2	It has led to longer patient waiting queues because of the time spent to apply all the stages of the IMCI case management protocol per under 5 patients.					
308.3	IMNCI is partially implemented because, if the IMCI trained nurses take too long assessing the under 5 patients, non-IMCI trained nurses take over care of other children waiting in the queue					
308.4	IMCI has reduced the number of follow-up visits by under 5 patients because of the thorough and accurate case management during initial visit.					
308.5	It is not practical to always refer to the IMCI chart booklet in the clinic during case management of every presentation.					
**308.6**	All IMCI trained nurses at our health facility apply all the stages of the IMCI protocol during case management of children under 5 years.					

308.7	Our health facility lay-out does not make it easy for us practice all the IMCI skills like witnessing first dose treatment, rehydration corners etc.					
308.8	The case management practices of IMCI trained nurses and non-IMCI trained nurses are inconsistent; hence caretakers of under5 prefer to be seen by nurses who are not IMCI trained.					

**309**	Which statement describes your experience in implementing the guidelines and procedures of the IMCI-1 strategy?					
		Strongly agree	Agree	Neutral	Disagree	Strongly disagree
309.1	IMCI is a user-friendly strategy for health workers					
**309.2**	IMCI is easy to understand and apply					
**309.3**	IMCI protocol is too long					
**309.4**	IMCI protocol is tedious					
**309.5**	IMCI is time-consuming					
**309.6**	IMCI is not practical to use at our health facility					
**309.7**	IMCI is difficult to understand and apply					
**309.8**	My supervisor does not appreciate rationale for IMCI					

**309.9**	My supervisor is not IMCI-trained					
**309.10**	Patient-nurse ratio does not allow for the use of IMCI strategy					
**309.11**	IMCI guidelines are too simplistic, it undermines my clinical training					
**309.12**	IMCI drugs are frequently out of stock					
**309.13**	Some clinical officers and doctors have negative attitudes towards IMCI					
**309.14**	IMCI wall charts and chart booklets are frequently unavailable					
309.15	Lack of IMCI follow-up training by IMCI facilitators					
309.16	Lack of supervision by IMCI trainers					
309.17	Our health facility is not fully equipped to support the use of be IMCI-strategy procedures					
309.18	Other (please specify :-)					
